# A novel co-drug of aspirin and ursolic acid interrupts adhesion, invasion and migration of cancer cells to vascular endothelium via regulating EMT and EGFR-mediated signaling pathways: multiple targets for cancer metastasis prevention and treatment

**DOI:** 10.18632/oncotarget.12232

**Published:** 2016-09-24

**Authors:** Qiao Tang, Yajun Liu, Tao Li, Xiang Yang, Guirong Zheng, Hongning Chen, Lee Jia, Jingwei Shao

**Affiliations:** ^1^ Cancer Metastasis Alert and Prevention Center, Pharmaceutical Photocatalysis of State Key Laboratory of Photocatalysis on Energy and Environment, College of Chemistry, Fuzhou University, Fuzhou, China; ^2^ Fujian Provincial Key Laboratory of Cancer Metastasis Chemoprevention, Fuzhou University, Fuzhou, China

**Keywords:** cancer metastatic chemoprevention, ursolic acid, aspirin, EMT, EGFR

## Abstract

Metastasis currently remains the predominant cause of breast carcinoma treatment failure. The effective targeting of metastasis-related-pathways in cancer holds promise for a new generation of therapeutics. In this study, we developed an novel Asp-UA conjugate, which was composed of classical “old drug” aspirin and low toxicity natural product ursolic acid for targeting breast cancer metastasis. Our results showed that Asp-UA could attenuate the adhesion, migration and invasion of breast cancer MCF-7 and MDA-MB-231 cells in a more safe and effective manner *in vitro*. Molecular and cellular study demonstrated that Asp-UA significantly down-regulated the expression of cell adhesion and invasion molecules including integrin α6β1, CD44, MMP-2, MMP-9, COX-2, EGFR and ERK proteins, and up-regulated the epithelial markers “E-cadherin” and “β-catenin”, and PTEN proteins. Furthermore, Asp-UA (80 mg/kg) reduced lung metastasis in a 4T1 murine breast cancer metastasis model more efficiently, which was associated with a decrease in the expression of CD44. More importantly, we did not detect side effects with Asp-UA in mice such as weight loss and main viscera tissues toxicity. Overall, our research suggested that co-drug Asp-UA possessed potential metastasis chemoprevention abilities via influencing EMT and EGFR-mediated pathways and could be a more promising drug candidate for the prevention and/or treatment of breast cancer metastasis.

## INTRODUCTION

Breast cancer is the second leading cause of cancer mortality worldwide [1]. Every year above 1.3 million women are diagnosed with breast cancer and nearly 450,000 women die from it. Invasion and metastasis, which is estimated to be responsible for approximately 90% of all cancer deaths [2, 3], is the primary factor that results in the failure of breast cancer treatment and poor prognosis. Traditional therapies of breast cancer include some surgery, radiation therapy and chemotherapy, among which chemotherapy still remains the backbone for treatment of metastatic breast cancer. However, traditional chemotherapy brings about severe side-effects and often lacks the ability to selectively target the cancer metastasis cascade, leading to distant relapse and repeated recurrence. Therefore, it is urgently needed to find safe and efficient cancer metastasis preventive agents for cancer survivors in this disease.

Metastasis is thought to begin with the epithelial–mesenchymal transition (EMT), a cascade of events in which tumor cells lose many of their ‘epithelial’ characteristics and become more like mesenchymal cells with the ability to spread and invade into the extracellular matrix (ECM) [4]. Extensive studies have shown EMT plays an important role in cancer metastasis [4–6]. The EMT process is very complicated and controlled by different signaling pathways, including cell adhesion molecules (CAMs), MMPs, COX, TGF-β, Wnt, MAPK, EGFR, PIP3 and others [7, 8]. Therefore, inhibiting EMT pathway of cancer cells as well as intervening with the key proteins in its related pathways should shed light on breast cancer progression and be informative for potential cancer metastasis chemopreventives.

Ursolic acid (UA), 3β-hydroxy-urs-12-en-28-oicacid, is an ursane-type pentacyclic triterpenic acid found in a variety of medicinal herbs and edible plants. It exhibits comprehensive biologic properties, such as anti-inflammatory [9], anti-angiogenesis [10], anti-cancer [11, 12] and liver protection [13], in a variety of human diseases. Recently, it has attracted considerable attention for its activities towards different cancers [14, 15]. In particular, it has the advantage of low toxicity, which makes it suitable for cancer metastatic chemoprevention. Prasad S *et al.* reported that ursolic acid can inhibit the metastasis of colorectal cancer through the suppression of multiple biomarkers linked to invasion, angiogenesis, and metastasis [16]. Jedinák A *et al.* found that UA significantly decreased the number of B16 colonies in the lungs of mice [17]. Our recent studies demonstrated that UA and its derivative US597, could safely and effectively suppress cancer metastasis both *in vitro* and *in vivo*, and are being developed as a novel cancer metastasis chemopreventive agent by us [18]. Various cellular targets and pathways have been proposed as to the mechanism of UA's anti-metastasis effects, including: integrin-mediated focal adhesion pathway [18], extracellular signal-regulated kinase (ERK) pathway [19], epidermal growth factor receptor (EGFR) [16] and MMPs [20] pathways, and suppressing several key molecules associated with cancer metastasis [21]. Overall, UA demonstrates extensive metastasis chemopreventive effects through multiple targets and pathways.

Aspirin (Asp), a non-steroidal anti-inflammatory drug (NSAID), is extensively used in clinical to treat rheumatism and prevent cardiovascular disease [22]. Recently, its therapeutic and prophylactic effects with regard to tumor metastasis are gaining widespread interest [23–26]. Maity *et al.* reported that aspirin could increase the expression of a set of markers, indicating a mesenchymal-to-epithelial transition (MET) in human breast carcinoma, which are most likely mediated through COX-dependent or independent pathways [27]. Jiang *et al.* showed that aspirin exerted anti-metastasis efficacy through inhibiting the expression of PI3K/AKT, ERK, NF-κB, CX3CL1, CD44 and MMPs [28, 29]. Very recently, we showed that a combination of aspirin and low toxicity drugs lysine, metapristone and doxycycline could prevent and treat tumor metastasis [30]. Therefore, aspirin also displays huge prospects in pre-metastatic chemoprevention.

In the present study, we firstly designed and synthesized a novel amphiphilic Asp-UA conjugate by combining the classical “old drug” aspirin and the natural anticancer product ursolic acid. The metastasis chemopreventive effect of Asp-UA co-drug on the adhesion-migration-invasion cascade of breast cancer cells were investigated at non-cytotoxic concentrations *in vitro*. Further, by applying multiple techniques including flow cytometry, immunohistochemistry and immunofluorescence, the underlying molecular mechanism of Asp-UA on the EMT and EGFR-mediated signaling pathways in MCF-7 and MDA-MB-231 cancer cells were explored. Additionally, we evaluated whether Asp-UA had a potential for the prevention or treatment of cancer metastasis *in vivo* by the 4T1 murine breast cancer lung metastasis model.

## RESULTS

### Synthesis and characterization of Asp-UA

**Figure 1:**
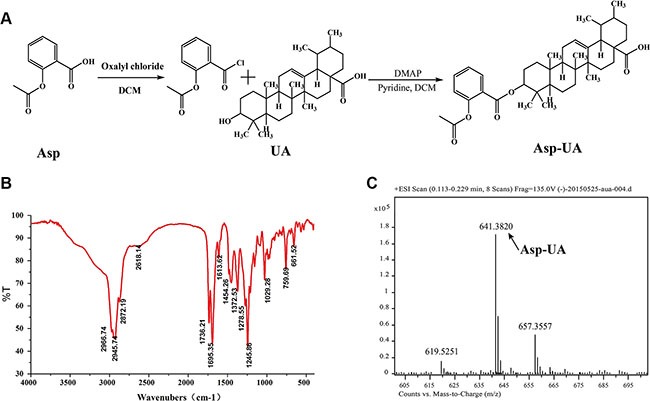
Synthesis scheme and spectral characterization of Asp-UA (**A**) The chemical synthesis route for Asp-UA. (**B**) The infrared spectra and (**C**) the mass spectra of Asp-UA.

To search for more safe and effective drug candidates for the prevention and treatment of cancer metastasis, we firstly synthesized the conjugate of the “old drug” aspirin and natural product UA. UA and Asp were used as the parent compounds and the structure modifications were performed at the C-28 position of UA. The synthesis route of conjugate Asp-UA was depicted in Figure 1. It was fully characterized by various spectroscopic methods, including infrared (IR), ^1^H-NMR and high resolution mass spectra (HRMS). IR and MS spectra were provided in Figure 1 and Supplementary Figure S1. UA was esterified to give Asp-UA of white powder with yield of 70.51%. Corresponding characterization of Asp-UA were as follows: 1H NMR (400 MHz, DMSO-d) d ppm 0.71−1.14 (m, 25 H) 1.18−1.38 (m, 4 H) 1.39−1.66 (m, 9 H) 1.74−1.95 (m, 5 H) 2.00 (s, 3 H) 2.11 (d, *J* = 11.29 Hz, 1H) 4.35−4.44 (m, 1 H) 5.13 (br. s., 1 H) 6.78−7.03 (m, 2 H) 7.44−7.58 (m, 1 H) 7.71−7.86 (m, 1 H) 11.95 (br. s., 1 H); HRMS [M+Na]^+^ calculated for C_39_H_54_O_6_Na, 641.3813, found, 641.3820. NMR spectra of Asp and Asp-UA with indicated peaks were illustrated in Supplementary Figure S2 and Supplementary Figure S3. Purity of Asp-UA was confirmed to be ≥ 95% by HPLC (Supplementary Figure S4).

### Effect of Asp-UA on cell viability

**Figure 2:**
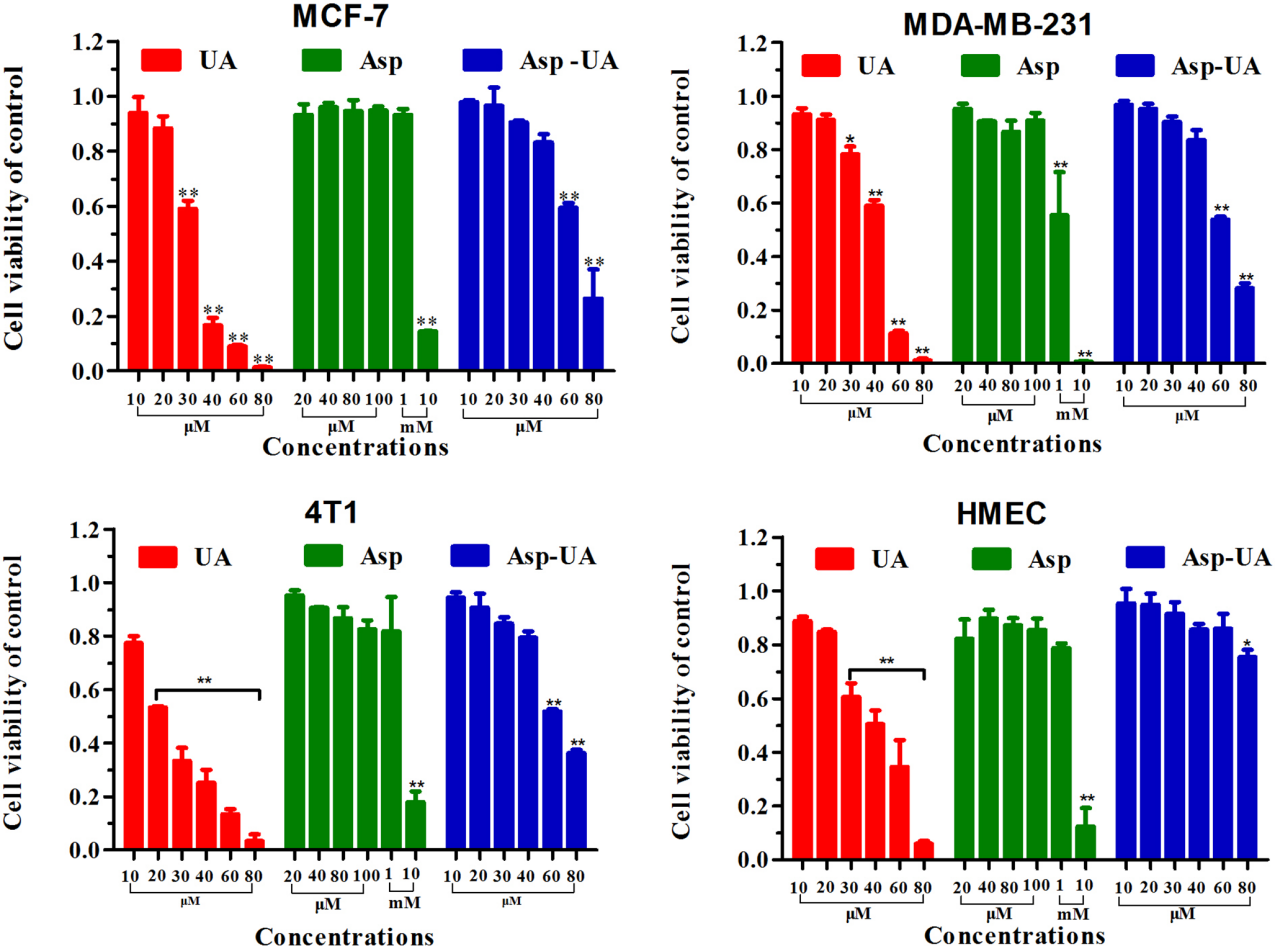
Inhibitory effects of UA/Asp/Asp-UA on the proliferation of human breast cancer MCF-7 and MDA-MB-231 cells, murine breast cancer 4T1 cells and the normal human mammary epithelial cell line HMEC The results shown were the mean of 3 parallel experiments for each concentration point. (*P < 0.05, **P < 0.01 in comparison with control).

To explore the metastasis chemoprevention function of Asp-UA, the cytostatic effects of the conjugate on different breast cancer cells and normal cells were firstly evaluated. The dose-dependent cell viabilities of four different cell lines treated with UA, Asp and Asp-UA for 24 hours were depicted in Figure 2. Asp-UA exhibited modest cytotoxic effects on human breast cancer cell lines MCF-7, MDA-MB-231 and murine breast cancer cell line 4T1 with an IC_50_ value of 72.16, 63.94 and 62.03 μM, respectively (Table 1). Free UA showed more suppressive cytotoxicity with IC_50_ values of 37.50, 42.32 and 23.33 μM, respectively (Table 1). Asp exerted negligible cytotoxicity at various tested concentrations and only exhibited certain cytotoxicity at mM concentrations (Table 1). In the mean time, Asp-UA inhibited normal human mammary epithelial cells (HMEC) viability at a much higher concentration with an IC_50_ value of >100 μM (Table 1). By comparison, the cytotoxicity of Asp-UA was very low at the concentration of 10~40 μM. Based on the cytotoxicity results, concentrations with negligible low toxicity (10–40 μM) were then chosen for further studies to explore the metastasis chemoprevention effects of Asp-UA *in vitro*.

**Table 1:** The cytotoxicity assay of the compounds towards different breast cancer cells vs normal cells

Compounds	IC_50_ (μM)			
	MCF-7	MDA-MB-231	4T1	HMEC
**UA**	37.50±2.32	42.32 ± 2.80	23.33 ± 0.42	39.27 ± 2.81
**Asp**	>1000	>1000	>1000	>1000
**Asp-UA**	72.16 ± 0.76	63.94 ± 0.79	62.03 ± 1.23	>100

Results expressed as IC_50_: half maximal inhibitory concentration. Data were obtained from 3 separate experiments and were mean ± SD.

### Effect of Asp-UA on the adhesion, invasion and migration of tumor cells *in vitro*

**Figure 3:**
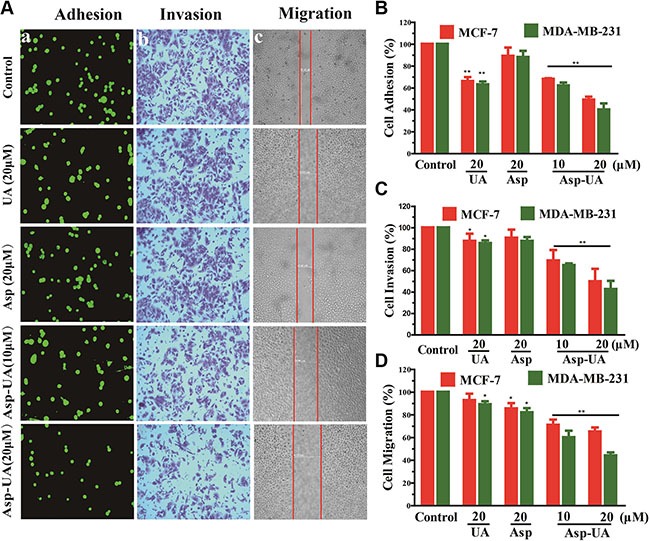
Effects of Asp-UA on metastasis of breast cancer cells *in vitro* (**A**) The adherent MCF-7 cells to ECM were photographed under the fluorescence microscope at ×200 magnification; b, Phase micrographs of invading MCF-7 cells were treated with UA, Asp or Asp-UA; c, Phase micrographs of MCF-7 cells were treated with UA, Asp or Asp-UA at 24 h after monolayer wounding. (**B**) Quantitative analysis of the inhibition by UA/Asp/Asp-UA on the adhesion of MCF-7 and MDA-MB-231 cells to matrigel. (**C**) Cells invaded through the membrane were quantified. (D) Migrated cells were quantified by manual counting. Data are obtained from 3 separate experiments and bars represent the mean ± SD. (*P < 0.05, **P < 0.01 in comparison with control).

The adhesion-dependent migration in tumor tissue is an important prerequisite for cancer cell dissemination [31, 32]. To determine whether Asp-UA affects the metastasis of breast cancer cells at non-cytotoxic concentrations *in vitro*, the cell-ECM adhesion, transwell and wound healing assay were performed to evaluate the adhesion, invasion and migration abilities of MCF-7 and MDA-MB-231 cells (Figure 3).

Within adhesion assay, we found that Asp-UA treatment for 1 h was able to significantly inhibit the adhesion of MCF-7 and MDA-MB-231 cells to matrigel in a dose dependent-manner (Figure 3A). In comparison with control group, the adhesion of MCF-7 cells to matrix was reduced by 32% and 51% (*p <* 0.01), respectively after treatment of 10 and 20 μM Asp-UA. Meanwhile, the adhesion of MCF-7 cells treated with 20 μM of UA were inhibited only by 34% (*p <* 0.05). Compared with the control group, the adhesion of MDA-MB-231 cells was reduced by 38% and 60% (*p <* 0.01) in 10 and 20 μM Asp-UA-treated groups, respectively. Interestingly, Asp alone exerts a slight effect in the tested concentrations (Figure 3A and Figure 3B).

Cell invasion is an important characteristic of malignant tumor cells compared with normal cells or benign tumor cells. To assess the ability of breast cancer cells to invade through matrigel, a transwell insert system was used to monitor the invasive process of breast cancer cells. As shown in Figure 3A, the average number of invaded MCF-7 cells was 488 ± 5, 426 ± 3 and 440 ± 6 in control, free UA, and Asp groups, respectively. However, in the Asp-UA treatment group of 20μM, only 242 ± 5 cells invaded through the monolayer membrane (Figure 3A). It's notable that under the same concentration of 20 μM, Asp-UA exhibited an inhibition rate of 50.32%, while UA and Asp showed 12.54% (*p <* 0.05) and 9.7%, respectively (Figure 3C). The inhibition efficiency of Asp-UA over free UA or Asp was evident with significant statistics difference. Moreover, using this matrigel invasion assay, the invasion capacity of MDA-MB-231, a highly aggressive breast cancer cell line, was also significant reduced by Asp-UA (Figure 3C).

The cell scratch assay demonstrated that the migration ability of MCF-7 and MD-MBA-231 cells in Asp-UA treated group was much lower than that in free UA, Asp or control group. The conjugate suppressed the migration in a significant dose-and-time-dependent manner in both two breast cancer cells (Figure 3A and Figure 3D). Furthermore, the migration assay showed that there were no significant inhibition effects in the two groups of UA and Asp, their effects were inferior to Asp-UA (Figure 3D). The above anti-metastasis results confirmed that Asp-UA was more potent than UA or Asp in inhibiting the adhesion, invasion and migration of breast cancer cells *in vitro*.

### Asp-UA down-regulates the mRNA and protein expression of cell adhesion molecules Integrin α6β1 and CD44 in MCF-7 and MDA-MB-231 cells

**Figure 4:**
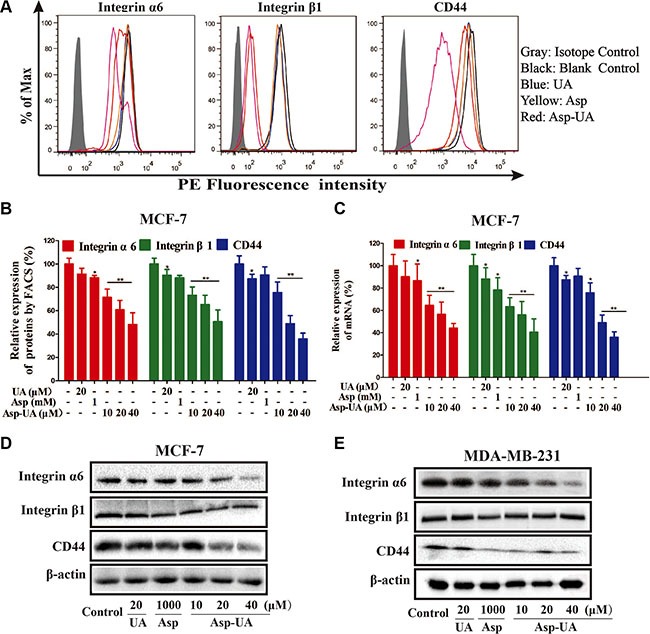
Influence of Asp-UA on the cell surface adhesion molecules (**A**) Expression of integrin α6, β1 and CD44 on MCF-7 cells was determined by flow cytometry, isotype control (gray area), control (black curve), blue curve (15 μM UA), yellow curve (1 mM Asp) and red line represents Asp-UA treated group at concentrations of 20 and 40 μM, respectively. (**B**) The inhibitory effects of UA/Asp/Asp-UA on the expression of integrin α6, β1 and CD44 by FACS. (**C**) qRT-PCR on MCF-7 cells. (**D**) The expression of integrin α6, β1 and CD44 by western blotting in MCF-7 cells. (**E**) MDA-MB-231 cells. Data are obtained from 3 separate experiments and bars represent the mean ± SD. (*P < 0.05, **P < 0.01 in comparison with control).

To verify the accurate molecular targets that Asp-UA affects the adhesion, invasion and migration of breast cancer cells, the expressions of various cell adhesion molecules (CAMs) on breast cancer cell surface were evaluated after exposure to UA, Asp, and Asp-UA, respectively. The protein expressions were quantitatively analyzed by flow cytometry. Among all the detecting proteins, we found integrin α6, β1 and CD44 proteins in MCF-7 and MDA-MB-231 cells were significantly down-regulated after treatment with Asp-UA for 24 h. As shown in Figure 4A, 4B and Supplementary Figure S5, when the MCF-7 cells were treated with 10, 20 and 40 μM Asp-UA, the fluorescence intensity of integrin α6 was inhibited by 28.55%, 39.24% and 51.76% respectively and β1 for 26.71%, 34.73% and 49.32%, respectively. Moreover, the Asp-UA treatment also caused a significant decrease in CD44 expression by 24.35%, 51.14%, and 64.19%, respectively (Figure 4A, 4B and Supplementary Figure S5). Meanwhile, similar trends were detected in another cell lines MDA-MB-231(results not shown), except for only a weaker effect for integrin β1. Under the same concentration, Asp-UA treatment was more efficacious than UA or Asp treatment alone. These results demonstrated that Asp-UA possessed better metastasis chemoprevention effects from the perspective of cell adhesion molecules (CAMs).

In addition, quantitative real-time PCR method (Figure 4C) also verified that mRNA levels of these CAMs were significantly reduced in MCF-7 cells. The results of qRT-PCR assay were similar with that obtained from the flow cytometry and western blotting analysis. The mRNA expression was significantly lower in the Asp-UA (40 μM) treated group than that in the control group (*P* < 0.01). UA and Asp also inhibited the expression of the corresponding mRNA, while the level of these mRNA showed no prominent change compared with Asp-UA.

We further detected the expression levels of integrin α6, β1 and CD44 by western blot method. As shown in Figure 4D and 4E, Asp-UA dose-dependently inhibited the expression of integrin α6, β1 and CD44 in MCF-7 cells (Figure 4D) and MDA-MB-231 cells (Figure 4E) after the Asp-UA treatment for 24 h. In MDA-MB-231cells, the inhibition effects were similar except for integrin β1, which has no obvious increase or decrease trend (Figure 4E). Meanwhile, a strong down-regulation of CD44 protein was detected in MCF-7 and MDA-MB-231 cells after the Asp-UA treatment for 24 h (Figure 4C and Figure 4E).

### Asp-UA down-regulates cell invasion molecules MMP-2, MMP-9 and COX-2 in MCF-7 and MDA-MB-231 cells

**Figure 5:**
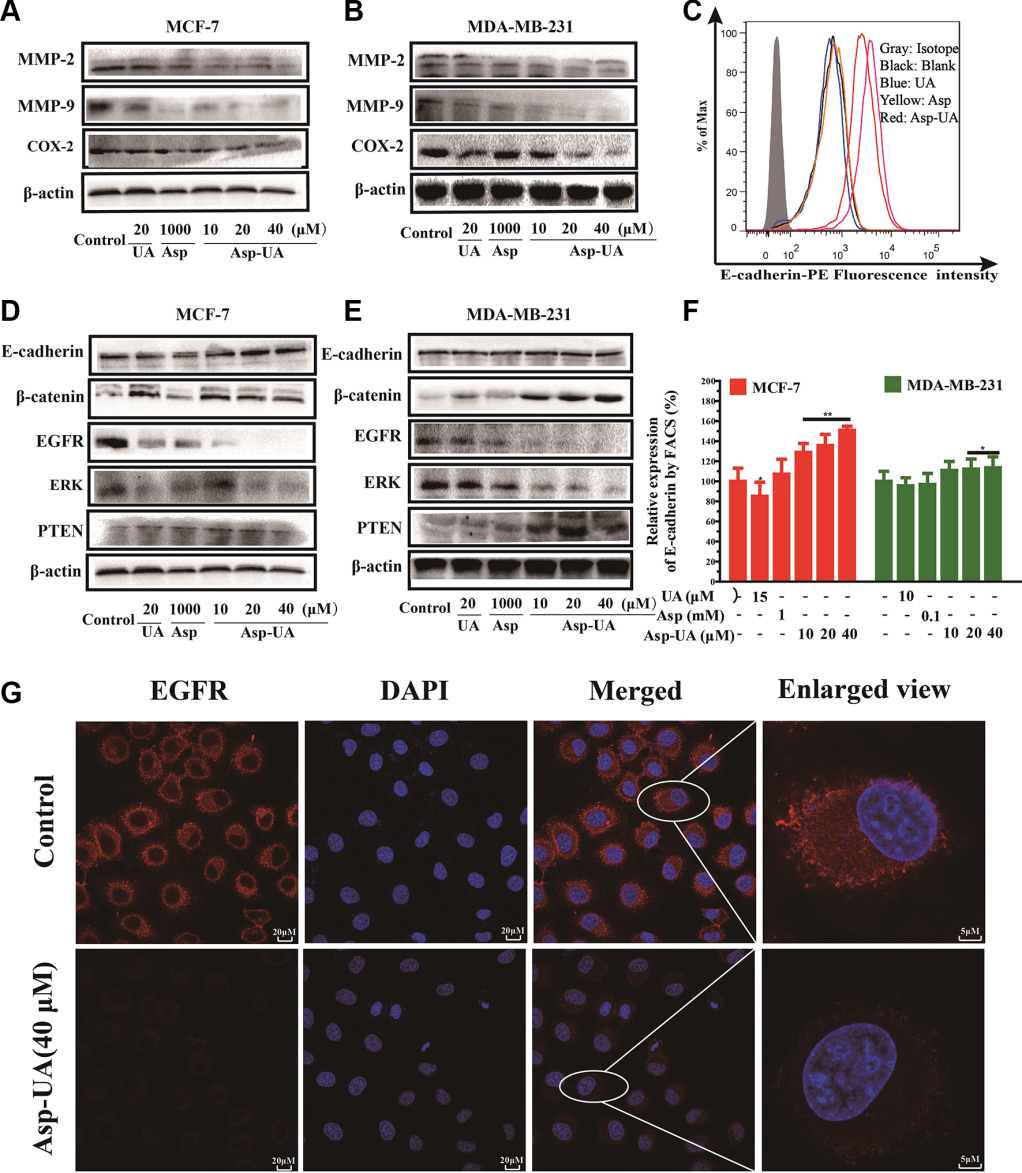
Influence of Asp-UA on the cell invasion molecules, EMT and EGFR related cell signaling pathways (**A**) Western blot analysis of UA/Asp/Asp-UA on the expression of MMP-2, MMP-9 and COX-2 in MCF-7 cells and (**B**) MDA-MB-231 cells. (**C**) Expression of E-cadherin on MCF-7 cells was determined by flow cytometry, isotype control (gray area), control (black curve), blue curve (15 μM UA), yellow curve (1 mM Asp) and red line represents Asp-UA treated group at concentrations of 20 and 40 μM, respectively. (**D**) Western blotting analysis of UA/Asp/Asp-UA on the regulation of E-cadherin, β-catenin, EGFR, ERK and PTEN in MCF-7 (**E**) and MDA-MB-231 cells (**F**). The inhibitory effects of UA, Asp and Asp-UA on the expression of E-cadherin by FACS. (**G**) Double immunofluorescence staining with DAPI (blue) and anti-EGFR antibody (red) was carried out in MCF-7 cells treated with Asp-UA for 24 h. The data were obtained from 3 separate experiments and bars represent the mean ± SD. (*P < 0.05, **P < 0.01 in comparison with control).

Due to the fact that Asp-UA could decrease the expression of CAMs, we further assessed the expression of crucial proteins including MMP-2, MMP-9 and COX-2, which are critical functional molecules within the tumor invasion process in breast cancer cells. As shown in Figure 5A and Figure 5B, Asp-UA (10, 20, 40μM) clearly reduced the expression levels of COX-2 and downstream MMP-2, MMP-9 proteins in MCF-7 and MDA-MB-231 cells in a concentration-dependent manner. In the Asp (1 mM) treated group, the expression of MMP-2 and MMP-9 proteins was obviously decreased, while the expression of COX-2 protein had no obvious change. Thus, Asp-UA can interfere with the invasion cascade by suppressing the crucial proteins of cancer invasion process.

### Asp-UA inhibits EMT-related signaling pathway

To investigate whether Asp-UA suppress EMT-related signaling pathway, we detected the relevant molecules in protein level with flow cytometry and western blot. FACS analysis (Figure 5C and Figure 5F) showed that 10, 20 and 40 μM Asp-UA treatment significantly increased E-cadherin expression by 28.72%, 35.64%, and 50.85%, respectively in MCF-7 cells. Furthermore, the WB results were consistent with FACS for E-cadherin in MCF-7 cells (Figure 5D). Interestingly, the expression of E-cadherin in MDA-MB-231 cells was very low, and the expression of E-cadherin was not up-regulated or down-regulated by the treatment of UA, Asp and Asp-UA (Figure 5E). We furtherly detected the β-catenin protein by western blot method. The expression of β-catenin significantly increased after Asp-UA treatment in MCF-7 cells (Figure 5D) and in MDA-MB-231 cells (Figure 5E). These results demonstrated that Asp-UA could interfere the tumor EMT-related process and therefore, further inhibit the metastasis of breast cancer.

### Asp-UA inhibits the EGFR-mediated signaling pathway in MCF-7 and MDA-MB-231 cells

To verify if Asp-UA can suppress EGFR-mediated signaling pathway, we detected several pivotal proteins in this signaling pathway by western blot assay. It turned out that Asp-UA could enhance the expression level of PTEN and reduce the expression levels of EGFR and ERK (Figure 5D and Figure 5E). Compared with UA or Asp, the regulation effects were remarkable when cells were treated with Asp-UA. Our results revealed that Asp-UA could inhibit EGFR-mediated pathway through increasing or decreasing the expression of proteins associated with cancer metastasis.

To confirm whether Asp-UA could inhibit the activity of EGFR, confocal imaging was performed to visualize its expression in MCF-7 cells. As a result, we found EGFR was mainly localized in the plasma membrane, with high expression in red fluorescence (Figure 5G) and the nucleus was stained in blue fluorescence with DAPI. However, the fluorescence intensity of EGFR was strongly reduced after treatment with Asp-UA (40 μM) for 24 h (Figure 5G).

### Effect of Asp-UA on experimental lung metastasis *in vivo*

**Figure 6:**
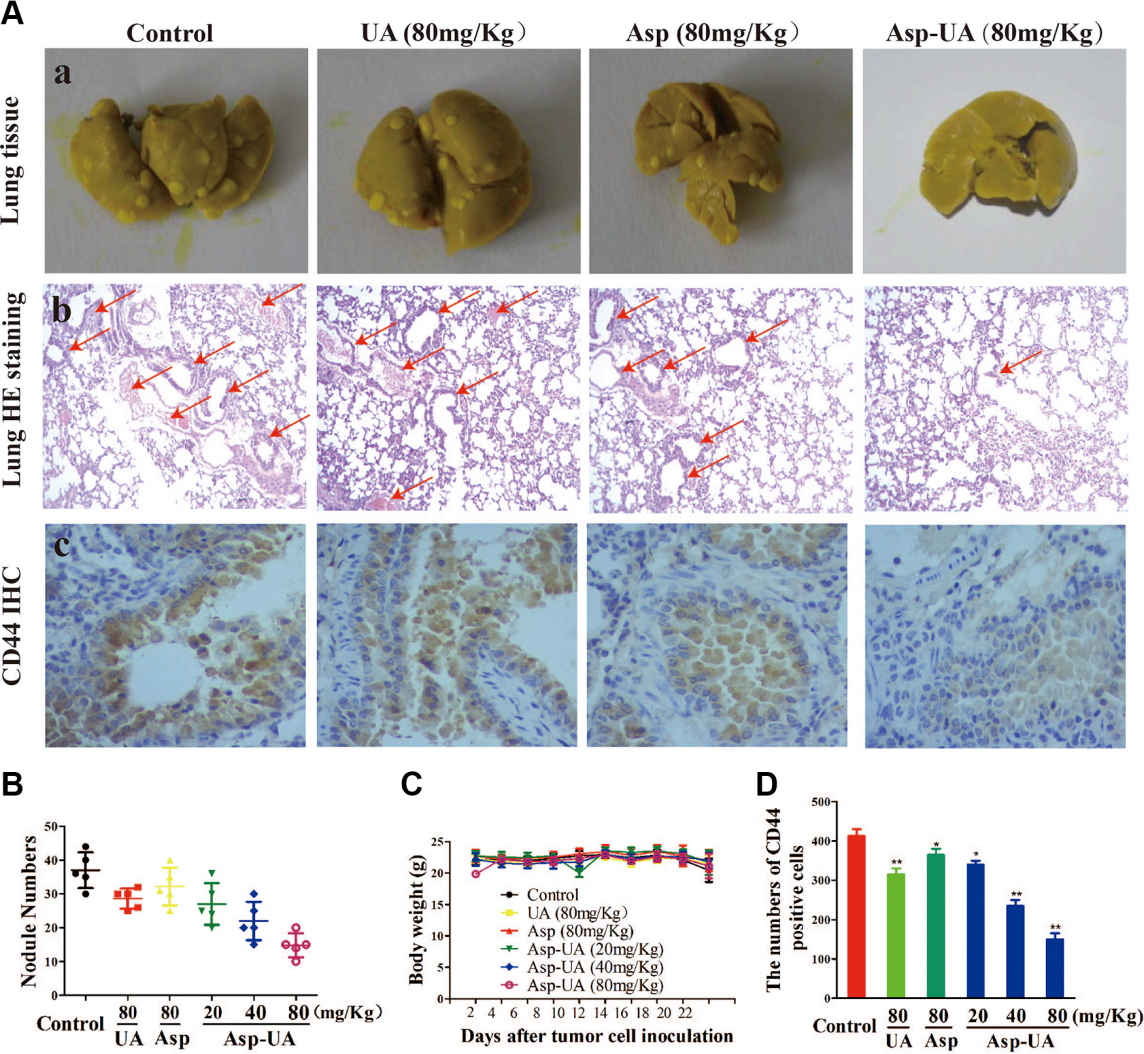
Effects of Asp-UA on metastasis of breast cancer *in vivo* (**A**) a, Photography of the lung of animals inoculated with 4T1 breast carcinoma cells via tail vein; b, Hematoxylin–eosin staining assay represent the metastasis in the lungs, amplification ×50; c, Immunostaining with antibodies to CD44 was performed on lung sections from control mice, UA/Asp/Asp-UA treated mice, original magnification ×200. (**B**) Metastatic tumor nodule numbers in lung metastasis model. (**C**) Body weight change during treatment periods. (**D**) Quantitative of the mean CD44 positive area counted at ×200. The results were expressed as mean ± SD. (*P < 0.05, **P < 0.01 in comparison with control).

To provide further evidence for the inhibition effect of Asp-UA on cancer metastasis *in vivo*, we examined the anti-metastatic efficacy of Asp-UA with a 4T1 murine artificial pulmonary metastatic model. The 4T1 cell line is a highly metastatic murine breast cancer with a proclivity for lung metastasis. As shown in Figure 6A (a) and Figure 6B, the number of metastatic foci at lung surfaces was significantly reduced in Asp-UA treated sets as compared with those of the other sets, supporting the assumption that Asp-UA may have anti-metastasis effect *in vivo*. In addition, the H&E staining further confirmed that the Asp-UA therapy reduced the metastatic tumor burden in the lungs in a disseminated breast cancer model (Figure 6A (b)).

**Figure 7:**
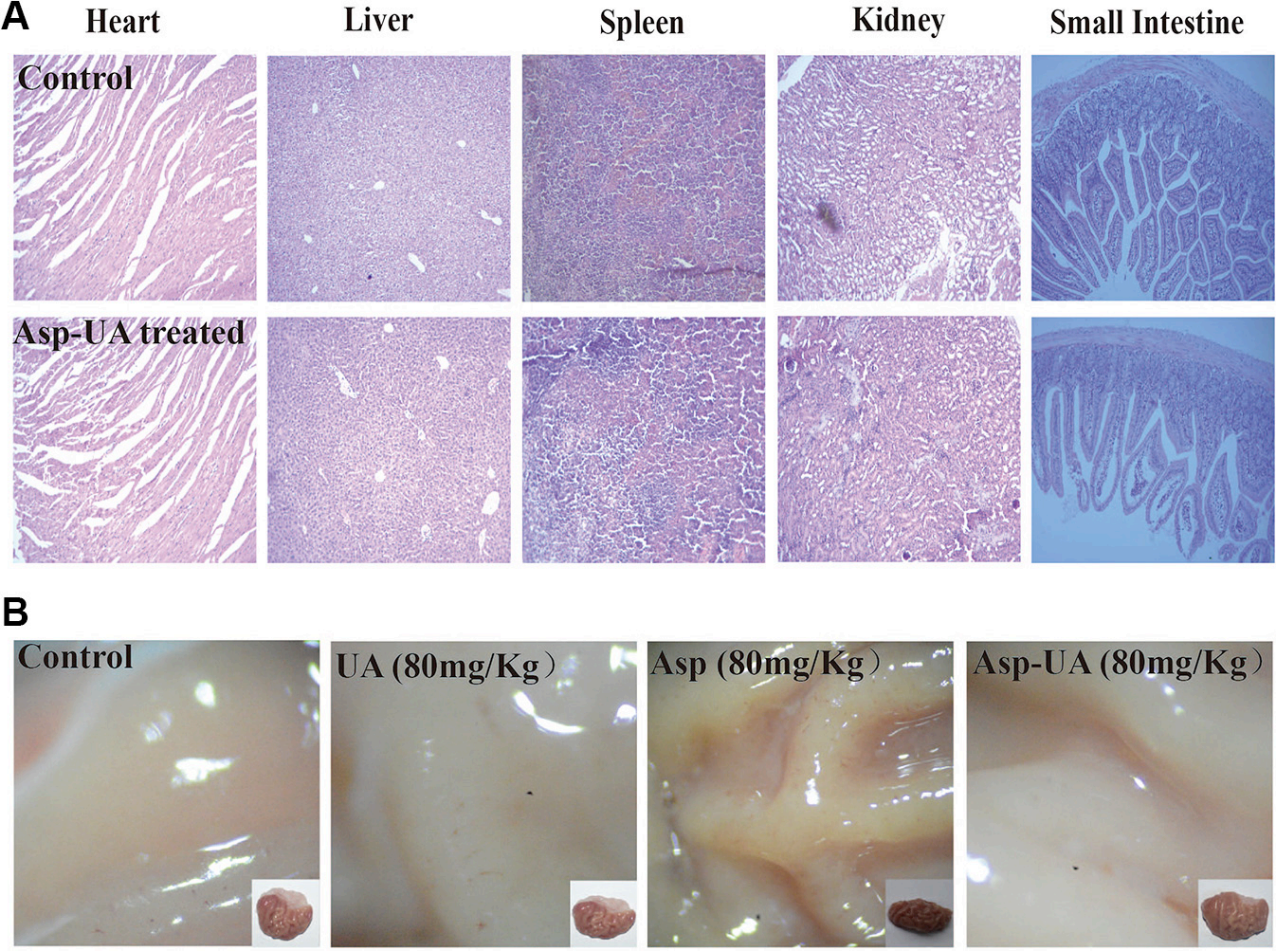
Main viscera tissues toxicity of Asp-UA in mouse and rat (**A**) H&E staining of paraffin-embedded sections of the heart, liver, spleen, kidney and small intestine. (**B**) The gastric mucosa of rats after orally gavage of UA/Asp/Asp-UA.

Meanwhile, Figure 6C depicted that there were no obvious body weight change in Asp-UA-treated groups (including 20, 40, and 80 mg/kg). Additionally, using H&E staining, histological sections of heart, liver, spleen, kidney and small intestine showed that Asp-UA didn't manifest any obvious toxicity as treated tissue sections appeared similar to that of control (Figure 7A). The main shortcoming of aspirin regimes is their gastrointestinal toxicity and the gastric mucosal toxicity assay showed that UA (80 mg/Kg) and Asp-UA (80 mg/Kg) didn't cause any congestion, corrosive focus, ulcer or lesions on the stomach tissue, while in Asp-treated group (80 mg/Kg) the prolapse of gastric mucosa and multiple bleeding points were observed (Figure 7B). Overall, these results suggested that the new compound Asp-UA didn't elicit obvious viscera tissues toxicity.

The above research clearly showed that the Asp-UA conjugate could suppress metastasis of 4T1 cells to the lungs, more potently than that of free UA or Asp alone. The therapy yielded significant anti-metastasis effects with no obvious toxicity in the 4T1 murine metastasis model. Therefore, it could be concluded that Asp-UA slowed the progression of breast cancer metastasis without observable adverse effects *in vivo*.

### Asp-UA inhibits the CD44 expression on the lung tissue of tumor metastatic mice

CD44 promotes migration and proliferation through interaction with many signaling molecules and it is a promising target for therapy in breast carcinoma. In order to observe the effects of Asp-UA on the expression of CD44 *in vivo*, the immunohistochemical staining method was used to analyze the expression of CD44 in lung tissues of metastatic mice. As shown in Figure 4, Asp-UA can efficiently down-regulate the expression of CD44 in breast cancer cells. Consistently, inhibition of CD44 expression in lung tissue was also detected by this IHC assay (Figure 6A (c)), which verified the effects of Asp-UA *in vivo*. Quantification analysis showed that the expression of CD44 was significantly decreased in Asp-UA treated group (Figure 6D). By comparison, a weaker inhibition was found in Asp or UA alone treated group.

## DISCUSSION

Breast cancer metastatic relapse accounts for 90% lethality of the disease. At present, plant materials possessing anti-metastasis activities are attractive candidates for anti-cancer agents. A vast amount of literature has documented the significant anti-angiogenesis, anti-adhesion and anti-invasion bioactivities of UAs, an important natural product and its derivatives [33–35]. Aspirin, the commonly used anti-pyretic and analgesic drug, has also demonstrated its anti-invasion and anti-angiogenesis activities [36]. However, a high dose of aspirin (~mM) is required to achieve the effect. Here we designed a new conjugate Asp-UA to combat cancer, especially targeting the cancer metastasis cascade. Conventional drug development on anti-cancer aims at seeking cytotoxic agents to kill cancer cells, which also brings tremendous damage to normal cells. Completely different from the traditions, our strategy was directed at blocking the initiation of the adhesion-invasion-migration cascade of cancer metastasis with the hypothesis that if tumor cells fail to adhere the endothelial layer or ECM, invade and migrate through the tumor surrounding tissue, they may lose part or all of their metastatic capacities. In our study, the results showed that Asp-UA effectively inhibited the migration, invasion and adhesion abilities of breast cancer cells *in vitro* in a low μM range (Figure 3), indicating a complementary synergistic effect of the ligation of two drugs.

Adhesion of cancer cells to ECM or vascular endothelium is a crucial starting point of metastasis [37]. These adhesion interactions are mediated by cellular adhesion molecules (CAMs), like integrin and cadherin [38], and the expression of these molecules play a major role in the adhesion process of tumor cells. We detected the expression of various CAMs on the surface of MCF-7 and MDA-MB-231 cells. Our flow cytometry results demonstrated that treatment of the two cell lines with Asp-UA could reduce the expression of integrin α6, β1, CD44 (Figure 4A and Figure 4B) and promote the expression of E-cadherin in MCF-7 cells (Figure 5C and Figure 5F), implying that integrin α6β1/CD44/E-cadherin on MCF-7 and MDA-MB-231 surface were partly responsible for the mechanism of Asp-UA on cancer metastasis prevention. The results also indicated that Asp-UA could block the tumor adhesion process via influencing the CAMs expressions.

The initial invasive action of metastatic cells involves interaction of tumor cells with the ECM, that is, cell matrix adhesion and then the ECM degradation. Accumulated studies have revealed that aspirin and UAs exhibit critical anti-metastatic effects via interference with MMP-mediated cell-matrix interaction [28, 39, 40–43]. In the present study, the effects of Asp-UA on the expression of MMP-2, MMP-9 and COX-2 proteins were determined. The results indicated that 10–40 μM Asp-UA effectively inhibited their expression in breast cancer cells in a dose-dependent manner (Figure 5A and Figure 5B). However, UA or Asp alone did not exhibit obvious inhibitory effect on the above-mentioned three proteins. This suggested that Asp-UA had a complementary synergistic effect on breast cancer metastasis by suppressing the expression of key proteins in breast cancer invasion.

Epithelial to mesenchymal transition (EMT) is an initial process which is associated with cancer metastasis [44]. EMT-related markers, such as E-cadherin and β-catenin, could sustain the epithelial phenotype and play critical roles in EMT transformation [45, 46]. In addition, extensive studies have revealed that high CD44 expression was correlated with EMT [47]. CD44 could disassociate the E-cadherin-β-catenin complex and release β-catenin into nucleus, leading the activation of genes related to cell invasion and migration [47]. The functions of these molecules have been reported by many researchers and their clinical potentials are very large due to their alert effects for early cancer metastasis [6, 48–50]. Our study demonstrated that Asp-UA was able to increase the expression of E-cadherin/ β-catenin, and decrease the CD44 expression (Figure 4 and Figure 6). The results suggested that Asp-UA effectively inhibited invasion and migration of breast cancer, maybe in part, via inducing significant changes of EMT-related proteins which then brought about the reversal of EMT.

**Figure 8:**
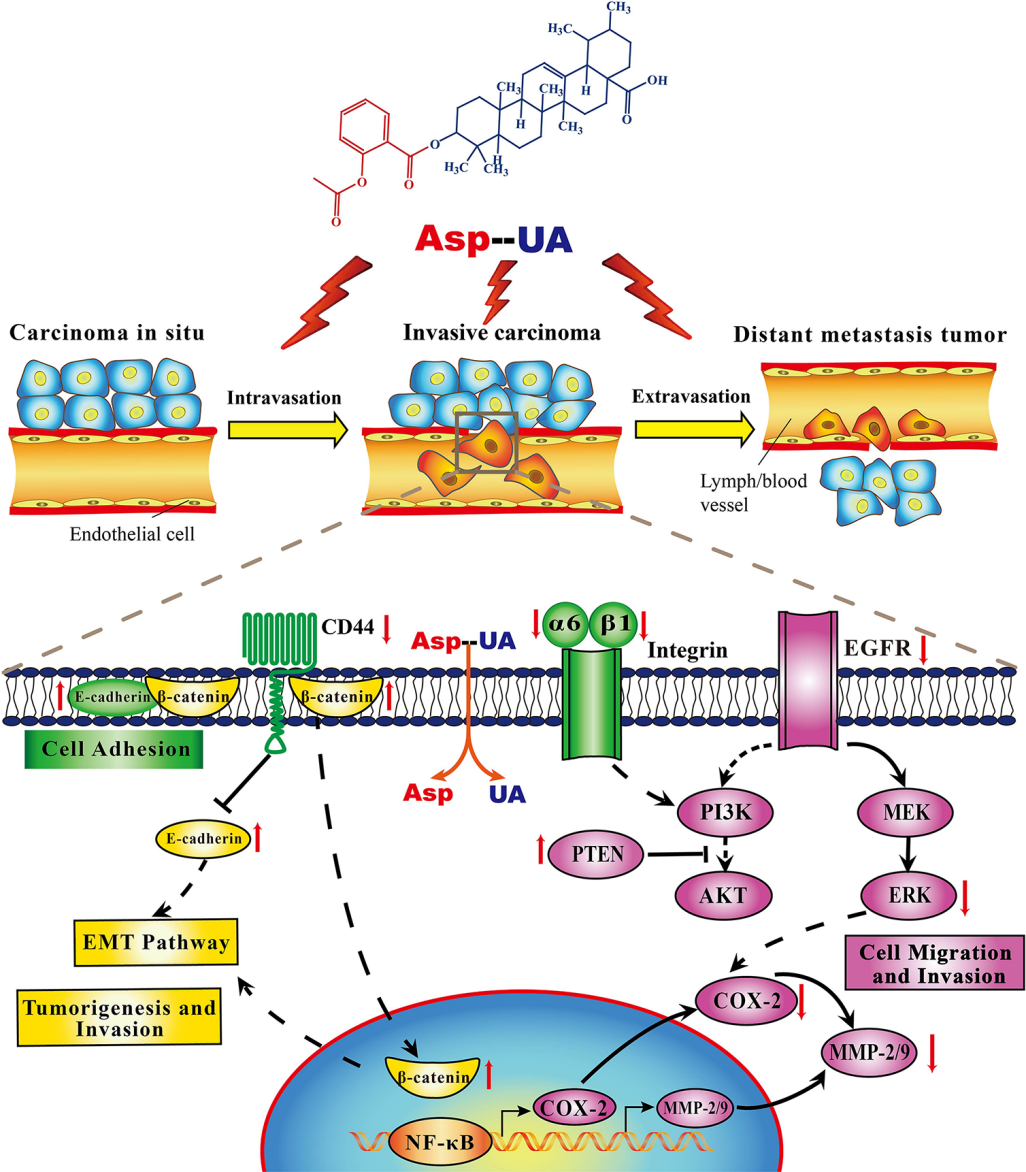
Proposed mechanisms diagram of signaling pathways for Asp-UA-regulated synergistic anti-metastasis

Activation and over-expression of EGFR play significant roles in the invasion and metastasis cascade of breast cancer. Prasad S *et al.* demonstrated that one of the mechanisms that UA used to inhibit metastasis was that UA might block the EGFR-mediated pathway within tumor cells [39]. In our study, we identified Asp-UA suppressed the expression of EGFR and ERK, and elevated PTEN expression level separately in Asp-UA-treated MCF-7 and MDA-MB-231 cells (Figure 5), which supported the fact that the inhibitory effects of Asp-UA on cell adhesion and invasion were partly associated with EGFR-mediated signaling pathway. All the above results showed that Asp-UA was able to modulate the expression of proteins involved in the metastasis cascade including adhesion, invasion and migration related molecules in breast cancer cells (Figure 8). Our data also suggested that Asp-UA may exert its metastasis chemoprevention effects by suppressing EMT and EGFR-mediated signaling regulator expression. The molecular research increases the understanding of the anti-metastatic mechanism of Asp-UA and provides evidence for the development of Asp-UA for the prevention and treatment of breast cancer metastasis.

To establish the relevance of these *in vitro* findings, the *in vivo* anti-metastasis effects of Asp-UA were further investigated in a mouse model of established pulmonary metastasis. We found the Asp-UA treatment resulted in remarkable reduction of lung metastasis (Figure 6A and Figure 6B), accompanied by significant down-regulation of the CD44 biomarker *in vivo*. In addition, we didn't detect obvious body weight change and significant abnormalities or lesions of main viscera tissues in Asp-UA-treated mice (Figure 6C and Figure 7). This observation demonstrated the application potential of Asp-UA in live systems. Our *in vivo* study also suggested Asp-UA possessed higher anti-metastasis efficacy with lower toxicity in comparison with UA or Asp alone. Oncologic therapeutic agent of high-efficiency and low-toxicity is an area of unmet clinical need [51]. The present study indicated that Asp-UA was effective in prevention and treatment of breast cancer metastasis, especially low toxic to the hosts, which could manifest a high clinical value. This kind of novel compounds could be combined with the current chemotherapeutic drugs or surgical approaches to have a profound therapeutic impact in the clinical applicaiton.

## MATERIALS AND METHODS

### Materials and reagents

Ursolic acid (UA, purity > 90%) was purchased from Xi'an Ocean Biological Engineering Co. (Xian, China). Aspirin (Asp, purity > 90%) was purchased from Aladdin Reagent Company (Shanghai, China). UA, Asp and Asp-UA were dissolved in dimethyl sulfoxide (DMSO) and were used in all experiments. PE-labeled mouse anti-human E-cadherin, vimentin, EpCAM and integrin α1, α3, α5, α6 and β1 integrin mAbs, PE/FITC mouse IgG1 kappa isotype control and APC mouse IgG1 kappa isotype control antibody were all purchased from Becton Dickinson (BD) Pharmingen^TM^ (New Jersey, USA). Mouse anti-human beta-actin (β-actin) antibody was purchased from Cell Signaling Technology (Danfoss, USA). Matrigel were obtained from BD Biocoat^TM^ (New Jersey, USA). The primary anti-EGFR, anti-ERK, anti-MMP-2, anti-MMP-9, anti-COX-2, anti-E-cadherin, anti-β-catenin, anti-integrin α6, anti-integrin β1, anti-PTEN and anti-CD44 antibodies were obtained from Abcam Biotechnology (Cambridge, UK). The secondary antibody goat-anti-mouse-IgG or goat-anti-rabbit-IgG horseradish peroxidase was obtained from Promega (Madison, USA). All other reagents used in this study were of the highest purity commercially available.

### Preparation of ursolic acid–aspirin conjugate

To a solution of 0.5 g Asp (2.78 mmol), 2 mL oxalyl chloride and one drop of dimethyl formamide in 20 mL dry dichloromethane was stirred at 25°C for 10 h. The reaction mixture was concentrated under reduced pressure to remove solvent. Then 20 mL dry dichloromethane was added in reaction mixture. Add a solution of 1.27 g of UA (2.78 mmol) and 0.1 eq DMAP in 20 mL of pyridine from the dropping funnel over a period of 30 minutes. The reaction mixture was stirred at 25°C for 10 h. After stirring, the reaction mixture was concentrated under reduced pressure to remove dichloromethane. 100 mL water was added and stirred at 25°C for 20 minutes. Then the reaction mixture was filtered to get crude product. The reaction mixture was purified on silica gel column using ethyl acetate: petroleum ether (v/v, 1:6) as eluents to give compound Asp-UA. All reactions were performed in oven-dried glassware. Yields refer to crystallised material or homogeneous products (TLC) obtained by column chromatography. All final compounds were purified to > 95% purity, as determined by a HPLC Breeze from Waters Co. using an Atlantis T3 5.0 μM 4.6 mm × 150 mm reverse phase column (Supplementary Figure S4).

### Cell lines and cell culture

Human breast cancer MDA-MB-231, MCF-7 cells, mouse 4T1 breast tumor cells and the normal human mammary epithelial cell line HMEC were purchased from the cell Bank of Shanghai Institute of Cell Biology. MDA-MB-231 and MCF-7 cells were cultured in L-15 and DMEM medium separately and the other cells were cultured in RPMI-1640 medium supplemented with fetal bovine serum (FBS, 10%), penicillin (100 U/mL) and streptomycin (100 μg/mL) in a humidified atmosphere of 5% CO_2_ at 37°C.

### Animals

Female BALB/C mice (age 6–8 weeks, body weight 20–25g) were purchased from Shanghai SLAC Laboratory. These mice were maintained with free access to pellet food and water in a controlled environment of 20–25°C, with lighting on from 6:00 AM to 6:00 PM. All animals used in these investigations were handled in accordance with protocol procedures, approved by the Institutional Animal Care and Use Committee of Fuzhou University. All efforts were made to minimize the animals suffering and to reduce the number of animals used.

### Cell viability measurement

The cytotoxicity of UA, Asp and Asp-UA was investigated by the MTT assay as previously described by this lab [52, 53]. Various cells including MCF-7, MDA-MB-231, 4T1 and HMEC were treated with UA, Asp or Asp-UA of indicated concentration for 24 hours. Cell viability was determined by detecting the absorbance at 570 nm using an infinite M200 Pro microplate reader (Tecan, Switzerland), triplicate wells were analyzed at each dose.

### Adhesion assay

The adhesion assay of MCF-7 cells to the matirgel was assessed according to the method described previously by this lab with minor modifications [18, 54]. Matrigel protein were diluted to appropriate concentration and pretreat 96-well culture plates with this matrigel for 12 h. Rhodamine 123-labeled MCF-7 cells were co-cultured with the matrigel protein in each well, followed by treatment with UA, Asp and Asp-UA for 1 hour. The non-adherent tumor cells were removed from the plate by washing with PBS, and the tumor cells bound to the matrigel were measured by fluorescence microscopy (Zeiss, Germany). The mean inhibition of adhesion for ten visual fields was calculated by using the equation: percent of control adhesion = [the number of adhered cells in treated samples/the number of adhered cells in the control group] × 100%.

### Invasion assay

Inhibition capacity against invasion of MCF-7 and MDA-MB-231 cells was estimated by transwell invasion assay as we described previously [18]. Briefly, in transwell cell culture chambers, 8 μM pore-sized transwell inserts in 24-well plate were coated with matrigel on the upper surface. The lower chambers were added with 750 μL of medium containing 20% FBS. MCF-7 or MDA-MB-231 cells were resuspended with reduced serum DMEM or L-15 medium and adjust the density to 2.5 × 10^5^/mL, and then cell suspension (200 μL) containing UA, Asp or Asp -UA were seeded onto the upper chamber wells. After 24 h incubation, the cells that had invaded through the matrigel membrane were fixed in methanol and stained with 0.1% crystal violet for 20 min. Soft remove the cells on the inner layer with a cotton swab. The filters were washed with PBS and images were taken. The invading cells were counted and photographed under a light microscope (Zeiss, Germany) at ×200 magnification. Five fields were counted per filter in each group and the experiment was conducted in triplicate.

### Migration assay

Scratch assay was performed to analyze cell migration *in vitro*, as described in our previous work [18]. The MCF-7 cells were seeded in 6-well plate; once confluent, a perpendicular scratch was generated in the surface of the plate using a pipette tip, followed by extensive washing with serum-free medium to remove cell debris, then incubated with DMEM medium containing 0.5% FBS and treated with or without UA, Asp and Asp-UA for 24 h and 48 h. Photographic images were taken from each well at indicated time after drug treatment, using a light microscope (Zeiss, Germany). The distance that cells migrated through the marked area was determined by measuring the wound width at 24 h/48 h after treatment, and comparing it with the wound width at 0 h. The experiment was repeated three times.

### Flow cytometry

This experiment was conducted as we described previously [55]. MCF-7 and MDA-MB-231 cells were grown on 6-well tissue culture plates followed by treatment with different concentration of UA, Asp and Asp-UA for 24 h. The cells were collected and incubated at 4°C for 30 min in the dark with the primary antibodies. Cell adhesion molecule E-cadherin, Vimentin, EpCAM and integrin (α1, α3, α5, α6 and β1) expression on cell-surface were measured by BD FACS AriaIII flow cytometry. The data were processed by FlowJo software and expressed as the mean fluorescent intensities.

### Western blotting analysis

Western blotting was performed as previously described [18]. Briefly, cell lysates were collected using immunoprecipitation (RIPA) lysis buffer. Samples with equivalent amounts of denatured proteins were resolved by SDS-PAGE using a 10% gel. After electrophoretic separation, proteins were transferred to a polyvinyldene difluoride (PVDF) membrane. The membranes were washed for ten minutes with TBS and were blocked by blocking solution. After washed the blots with TBST for three times, membrane was probed with the primary antibody diluted 1:1000 in TBST solution overnight at 4°C, followed by incubation with a secondary antibody conjugated with horseradish peroxidase diluted 1:5000 in TBST solution for 2 h at37°C. After the membranes were washed with TBST and TBS, the proteins of interest were detected by using SuperSignal West Pico Chemiluminescent Substrate kit and detected by using the ChemiDoc XRS system (Bio-Rad). The target proteins expression was quantified by use of Image Lab analysis software (Bio-Rad).

### Total RNA isolation and quantitative real-time PCR

Total RNA was isolated from cells using TRIZOL Reagents (Invitrogen). A sample (1 μg) of total RNA was used to generate cDNA templates by RT reaction with the PrimeScript^®^ RT reagent Kit (Takara, Dalian, China) according to the manufacturer's instructions. The first strand cDNA products were used as PCR templates and the PCR amplificationwas performed in a 25 μL reaction volume using a SYBR^®^Premix Ex Taq™ PCR kit (Takara, Dalian, China), using SYBR-Green real-time PCR method on the CFX96TM Real-Time PCR Detection Systems (Bio-Rad). The sequences are 5′-TCCCTGAA CCTAACGGAGTCT-3′ and 5′-ATGTCCAAGTAGTTCA GTTTG-3′ for integrin α6, 5′-ACAGCAGAGAAGCTGA AGCCA-3′ and 5′-GAGCTTAGCTGGTGTTGTGC-3′ for integrin β1, 5′-AGACGAAGACAGTCCCTGGATCAC-3′ and 5′-TGTGTTTGCTCCACCTTCTTGACTC-3′ for CD44, 5′-TGGCACCCAGCACAATGAA-3′ and 5′-CATA GTCATAGTCCGCCTAGAAGCA-3′ for β-actin. Target mRNA expression levels were normalized with the β-actin mRNA and the relative changes of gene expression were measured using the 2−ΔΔCT method. Results are expressed as the mean ± standard deviation of three independent experiments.

### *In vivo* tumor growth and metastasis assay

Subconfluent 4T1 cells were harvested and resuspended in PBS to a final density of 1 × 10^5^ cells/mL. Then inject 200 μL into the tail vein of female BALB/C mice. On the following day (Day 1), mice were randomly divided into six groups (*n* = 8 for each group) and then normal solvent (CMC-Na (control)), UA (80 mg/kg), Asp (80 mg/kg) and Asp-UA (20, 40 and 80 mg/kg), respectively, were administered to mice for 28 days via orally gavage. Mice weight were measured every other day. After the treatment, the mice were sacrificed by cervical dislocation after euthanizing with isoflurane (3%). The heart, liver, spleen, lung, kidney and small intestine were dissected, washed with PBS and fixed in 10% (v/v) buffered formaldehyde. These tissues were then paraffin embedded and stained with hematoxylin and eosin (H&E). The number of surface tumor nodules was counted visually with the aid of a dissecting microscope and histological observations were performed under a microscope (Zeiss, Germany).

### Rat gastric mucosal injury

On the first day (Day 1), mice were randomly divided into six groups (*n* = 8 for each group) and then normal solvent (CMC-Na (control)), UA (80 mg/kg), Asp (80 mg/kg) and Asp-UA (20, 40 and 80 mg/kg), respectively, were administered to mice for 7 days via orally gavage. After the treatment, the mice were sacrificed by cervical dislocation after euthanizing with isoflurane (3%). Cut the stomach across the greater curvature and expose the gastric mucosal, followed by washing with PBS and examination under a light microscopy.

### CD44 immunohistochemistry

Frozen sections with the thickness of 6μm were stained with anti-CD44 antibody as previously described [48]. The presence of the CD44 protein was visualized by DAB staining and examined under a microscope. Results for CD44 positive cells are shown as ×200 magnification. Positive signals of CD44 IHC showed brow-yellow cytoplasmic staining. To quantitatively examine the number of CD44 positive cells, ten fields from each tissue section were randomly selected, photographed and analyzed with the MIQAS analysis system (Biological technology Corp., Shanghai, China).

### Immunofluorescence microscopy

MCF-7 cells grown on 35 mm cell culture dish with 10^5^ cells were co-cultured with different drugs and then rinsed three times using PBS. Subsequently, the cells were fixed with fresh 4% paraformaldehyde for 30 min. The cells were then washed three times with PBS, and permeabilized with 0.1% Triton X-100 for 5min and then blocked with fresh 10% goat serum followed by incubation with rabbit anti-EGFR (1:100) for 12 h in 4°C. The cells were then rewashed, and incubated with CY3-conjugated goat anti-rabbit IgG (Boster, BA1032) for 1 h in room temperature. Finally, the cells were washed and restained with DAPI. Blue fluorescence of DAPI, red fluorescence of EGFR was observed using a Zeiss LSM 780 confocal microscope (Zeiss Co., Germany).

### Statistical analysis

Data were presented as the means ± standard deviations (SD) for three independent experiments. Statistical analysis was done by student's *t-test* and one-way analysis of variance using least significance difference test (IBM SPSS Statistics 19.0). A probability (*P*) value < 0.05 was considered statistically significant, and P < 0.01 was highly statistical significance.

## CONCLUSIONS

In the present study, for the first time, we demonstrated that a novel co-drug Asp-UA had better inhibitory effect on breast cancer metastasis and low toxicity both *in vitro* and *in vivo* compared with UA and Asp alone. Asp-UA managed to significantly inhibit the adhesion-invasion-migration cascade of MCF-7 and MDA-MB-231 cells by down-regulating the relevant functional molecules. These effects were accompanied by suppressing the EMT and EGFR-mediated pathways. Moreover, Asp-UA also undermined the progression of pulmonary metastasis for 4T1 cells in female BALB/C mice without obvious side effects such as weight loss and main viscera tissues. Taken together, this newly-discovered activity of Asp-UA makes it best-suited for the development of chemo-preventive drugs for cancer metastasis. Our results will promote better use of existing drugs and facilitate the design of more complex therapeutic approaches to target advanced malignancies, and more importantly, provide a molecular basis for clinical evaluation of Asp-UA as a potential cancer metastatic chemopreventive agent.

## SUPPLEMENTARY MATERIALS FIGURES


