# Supplementary material for: A novel co-drug of aspirin and ursolic acid interrupts adhesion, invasion and migration of cancer cells to vascular endothelium via regulating EMT and EGFR-mediated signaling pathways: multiple targets for cancer metastasis prevention and treatment
Source: Oncotarget. 2016 Sep 24;7(45):73114–29. doi: 10.18632/oncotarget.12232 (PMC5341967; doi:10.18632/oncotarget.12232)
Supplement: Supplementary file 1 [file oncotarget-07-73114-s001.pdf]

A novel co-drug of aspirin and ursolic acid interrupts adhesion, invasion and migration of cancer cells to vascular endothelium via regulating EMT and EGFR-mediated signaling pathways: multiple targets for cancer metastasis prevention and treatment

Supplementary Materials

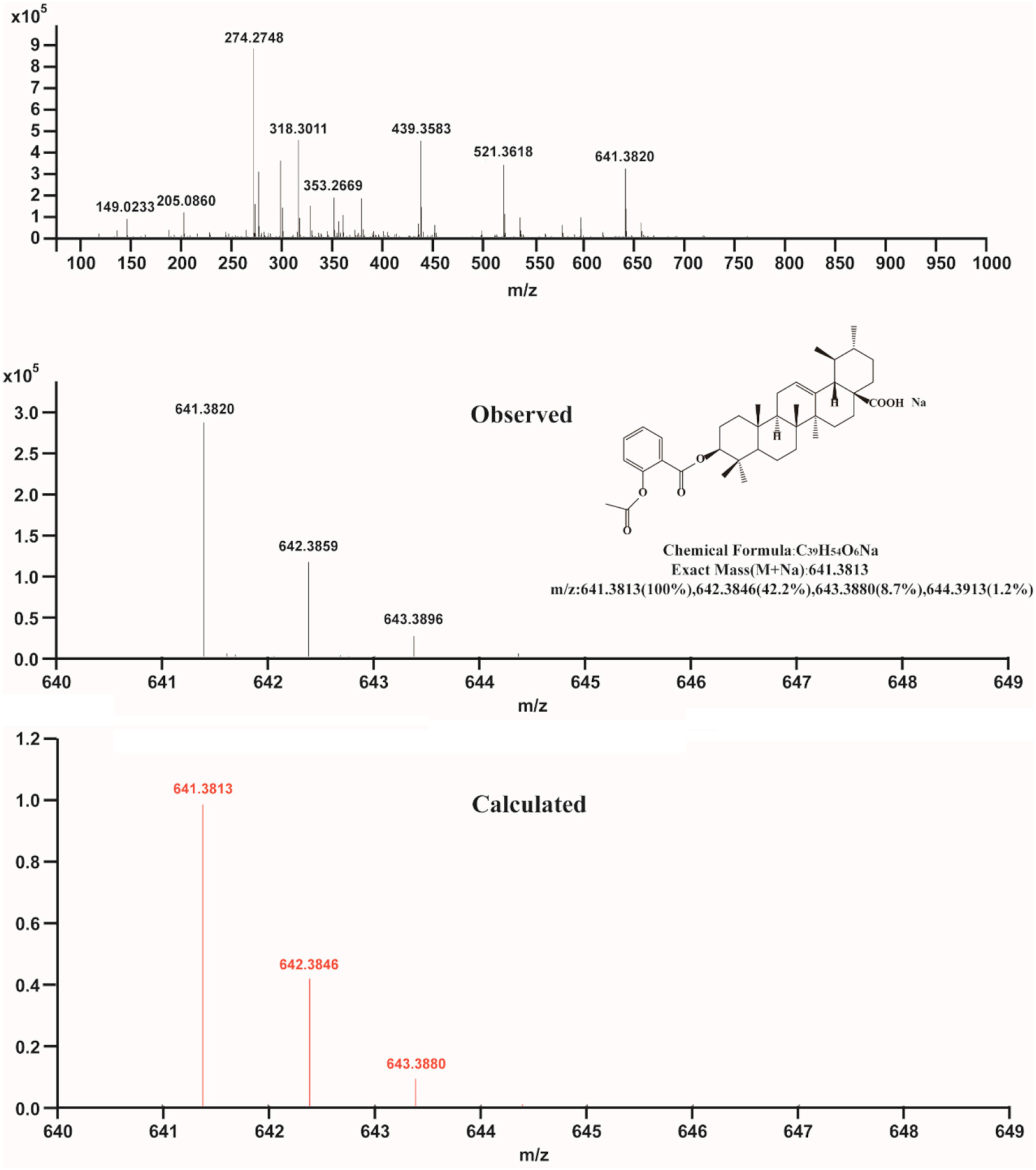

Supplementary Figure S1: ESI-HRMS spectra of Asp-UA.

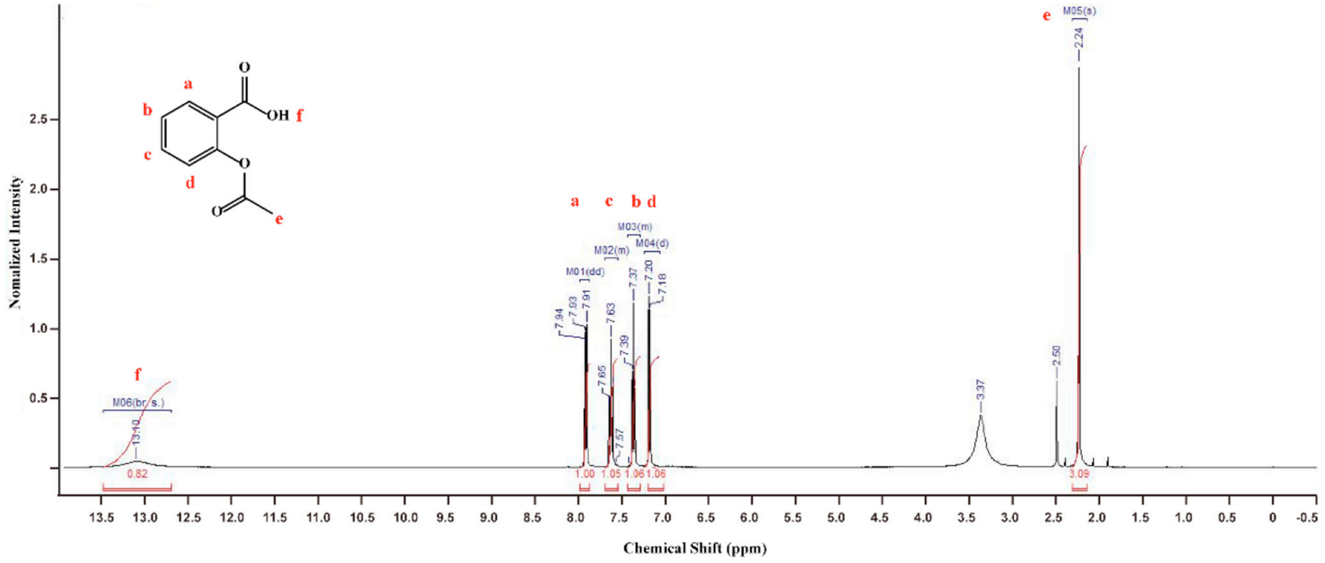

Supplementary Figure S2: NMR spectra of Asp with indicated peaks (400 MHz, DMSO-d_6, δ, ppm).

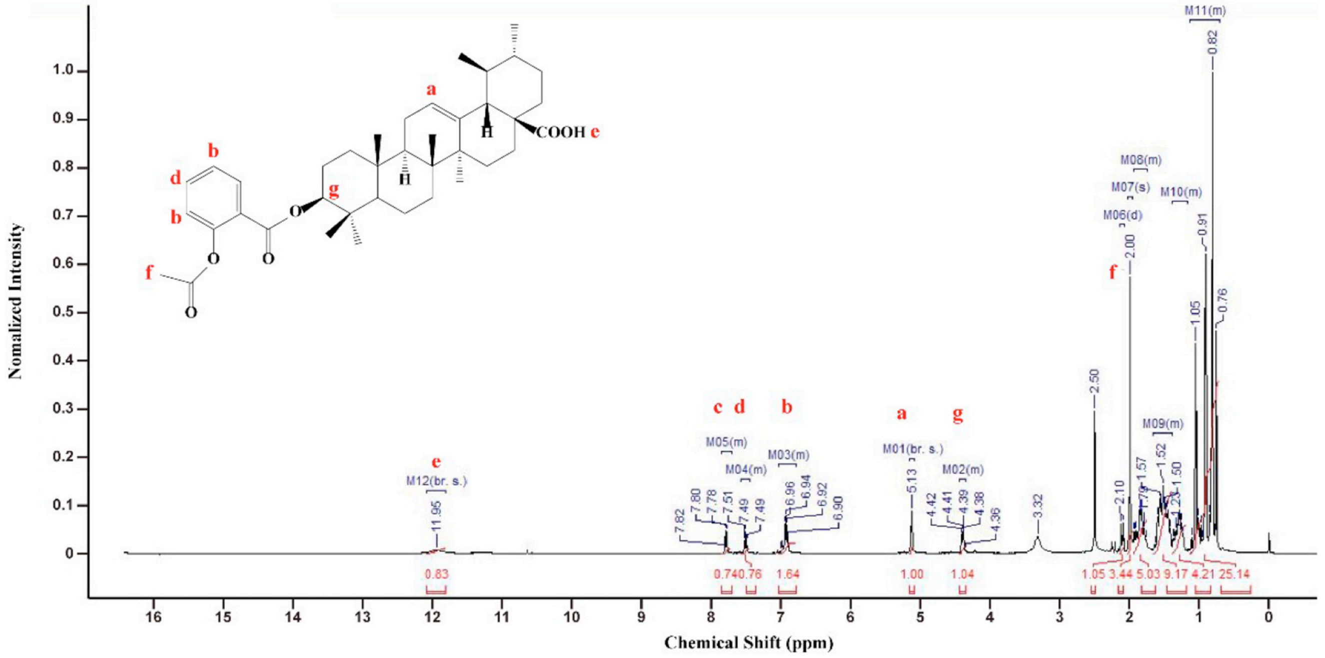

Supplementary Figure S3: NMR spectra of Asp-UA with indicated peaks (400 MHz, DMSO-d_6, δ, ppm).

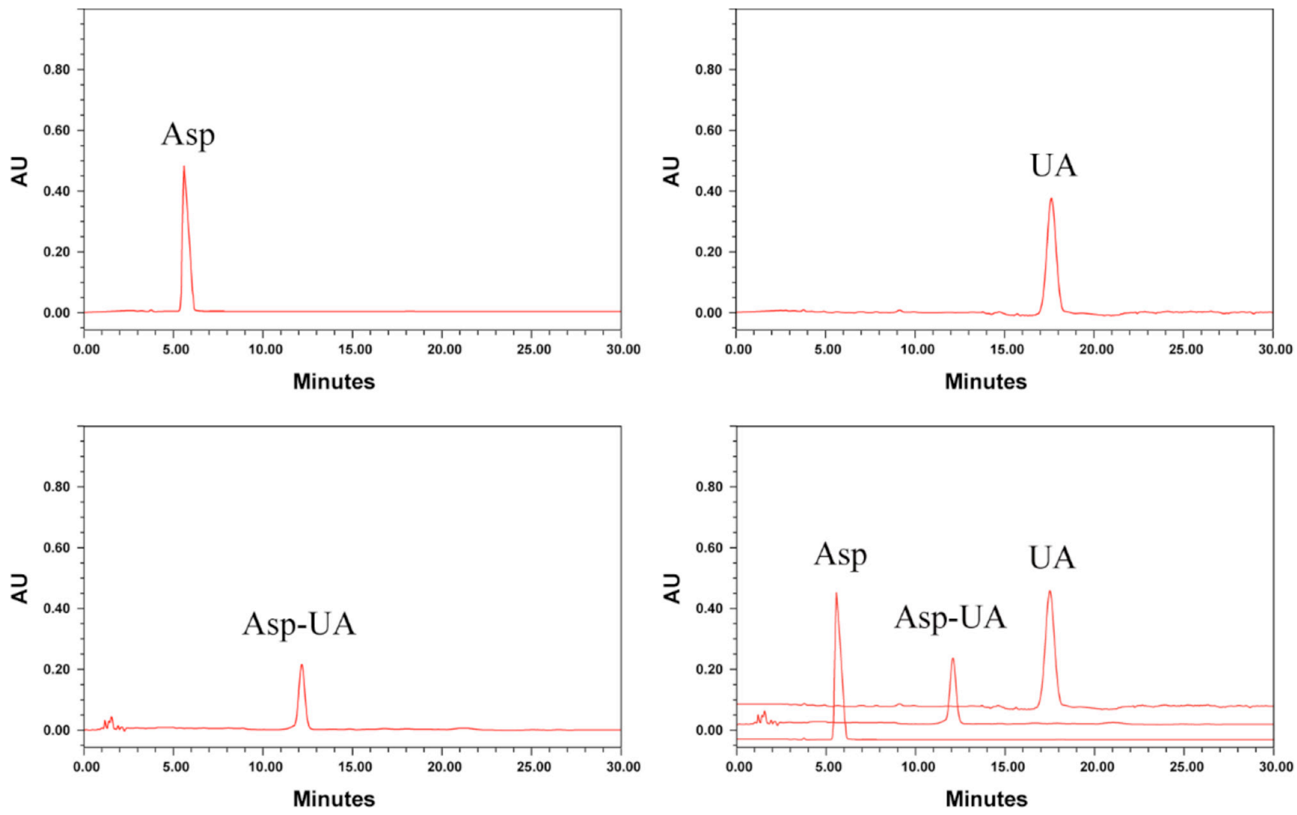

Supplementary Figure S4: HPLC analysis on the reaction products of Asp-UA. Pure standard aspirin and ursolic acid were used for comparison. Wavelength used: 280 nm.

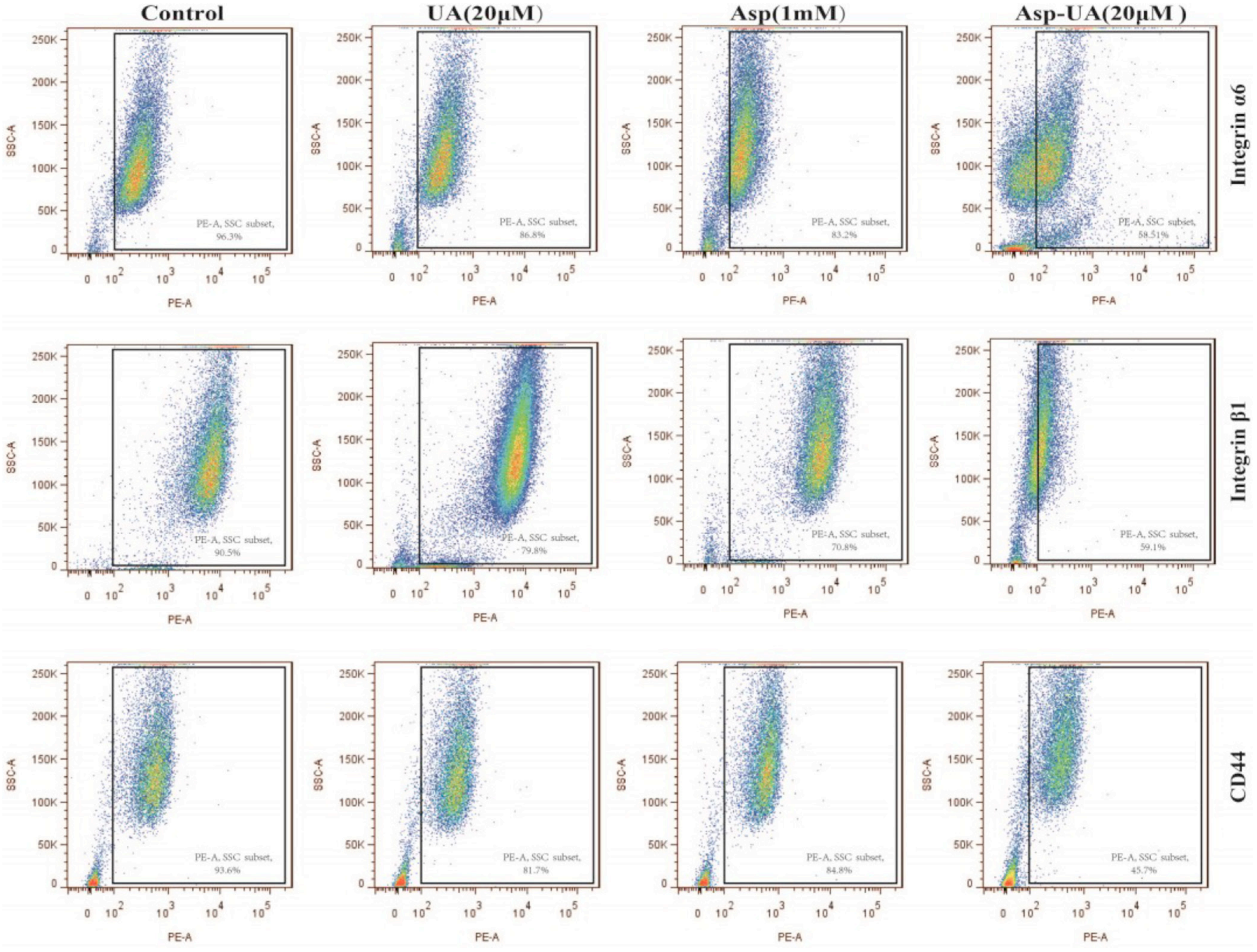

Supplementary Figure S5: FACS analysis on the expression of integrin α6, β1 and CD44 by UA/Asp/Asp-UA treatment with results in dot plot format.
